# Gut microbiota of Parkinson’s disease in an appendectomy cohort: a preliminary study

**DOI:** 10.1038/s41598-023-29219-2

**Published:** 2023-02-07

**Authors:** Keiichi Nakahara, Shunya Nakane, Kazuo Ishii, Tokunori Ikeda, Yukio Ando

**Affiliations:** 1 Department of Neurology, Graduate School of Medical Sciences, Kumamoto University, 1-1-1 Honjo, Chuo-Ku, Kumamoto, 860-8556 Japan; 2 Department of Neurology, Nippon Medical School Hospital, 1-1-5 Sendagi, Bunkyo-Ku, Tokyo, 113-8603 Japan; 3 Biostatistics Center, Kurume University, 67 Asahi-Machi, Kurume, Fukuoka 830-0011 Japan; 4 Department of Clinical Investigation, Kumamoto University, 1-1-1 Honjo, Chuo-Ku, Kumamoto, 860-8556 Japan

**Keywords:** Microbiology, Diseases, Gastroenterology, Neurology

## Abstract

In patients with Parkinson’s disease (PD), α-synuclein pathology is thought to spread to the brain via the dorsal motor nucleus of the vagus nerve. The link between the gut microbiome and PD has been explored in various studies. The appendix might play an important role in immunity by maintaining the microbiota as a reservoir. In recent times, appendectomy has been linked to a lower risk of PD, possibly owing to the role of the appendix in altering the gut microbiome. We aimed to elucidate whether the gut microbiota affects PD development in the appendectomy cohort. We analyzed the fecal microbial composition in patients with PD and healthy controls with and without a history of appendectomy. The abundance of microbes from the family *Enterobacteriaceae* was higher in feces samples from patients with Parkinson’s disease compared to that in samples collected from healthy controls. Furthermore, there was a significant phylogenetic difference between patients with PD and healthy controls who had undergone appendectomy. There was a significant phylogenetic difference between patients with PD and HCs who had undergone APP. These results suggest the correlation between gut microbiota and PD in patients who have undergone APP.

## Introduction

Parkinson’s disease (PD) is the most common neurodegenerative movement disorder. It is neuropathologically characterized by the presence of Lewy bodies composed of α-synuclein. α-synuclein pathology spreads to the brain via the dorsal motor nucleus of the vagus nerve^1^. Although α-synuclein aggregates in the vermiform appendix in nearly all individuals, including patients with PD as well as individuals without any neurological disease, the abundance of α-synuclein in the insoluble fraction from the appendix is higher in patients with PD compared to that in healthy controls (HCs)^2^.

The gut microbiota plays a significant role in the development and homeostasis of different body systems, including the central nerves system (CNS), via the gut-brain axis^3,4^. The fecal microbiome of patients with PD has been shown to differ from that of controls^5,6^.

The appendix might play an important role in immunity by maintaining the microbiota as a reservoir^7^. The gut microbiota may affect the dissemination of insoluble α-synuclein aggregates from the vermiform appendix to the brain via the vagal nerve. To elucidate the correlation between microbiota and PD in the appendectomy (APP) cohort, we analyzed the fecal microbial composition in patients with PD and HCs with and without a history of APP. This is a preliminary study, but the findings of the present study indicate a meaningful association between the altered gut microbiota and the role of the appendix in the pathogenesis of PD.

## Results

### Demographic and clinical features of participants

The median age of the participants was 70.0 years (interquartile range 67.0–71.0 years). The majority of the patients were female (60%). Patients with PD tended to have a more lean body structure compared to HCs, and patients with PD, particularly those with PD/APP+, had more severe constipation, but none of these differences were statistically significant (*p*-value = 0.120, and 0.219, respectively, using the Kruskal–Wallis test). No significant differences were observed in the age at onset, disease duration, Hoehn & Yahr (HY) scale, Unified Parkinson’s Disease Rating Scale (UPDRS), mini-mental state examination (MMSE), Odor Stick Identification Test for Japanese (OSIT-J), and levodopa-equivalent daily dose (LEDD) between the PD/APP+ and PD/APP− groups (Table 1).

**Table 1:** Demographic and clinical features.

	Healthy controls		PD patients	
	APP	No APP	APP	No APP
Number of participants	5	5	5	5
Male (%)	2 (40)	2 (40)	2 (40)	2 (40)
Age, year, median IQR	70.0 (62.0, 76.5)	69.0 (59.0, 71.5)	70.0 (63.0, 77.5)	70.0 (67.0, 71.0)
Age at onset, year, median IQR	–	–	67.0 (59.5, 74.5)	66.0 (64.0, 68.5)
PD duration, year, median IQR	–	–	3.0 (3.0, 3.5)	2.0 (1.5, 5.0)
Height, cm, median IQR	160.0 (147.9, 165.0)	159.0 (152.0, 165.0)	159.6 (153.5, 160.5)	155.0 (154.0, 167.5)
Weight, kg, median IQR	62.0 (55.5, 68.5)	57.0 (52.0, 74.0)	59.5 (40.6, 60.7)	55.0 (52.0, 67.0)
BMI, kg/m^2^, median IQR	24.6 (23.7, 26.5)	23.6 (21.6, 27.8)	23.4 (17.3, 23.6)	22.3 (21.6, 24.8)
HY scale, median IQR	–	–	2.0 (1.5, 2.5)	2.0 (1.5, 2.5)
UPDRS, median IQR				
Total	–	–	26.0 (13.5, 35.0)	24.0 (15.0, 29.5)
Part 1	–	–	1.0 (0.5, 2.0)	1.0 (1.0, 3.5)
Part 2	–	–	9.0 (4.0, 11.5)	5.0 (2.5, 8.5)
Part 3	–	–	12.0 (7.0, 19.5)	13.0 (10.5, 18.0)
Part 4	–	–	2.0 (1.0, 3.5)	1.0 (0.5, 2.0)
MMSE, median IQR	–	–	30.0 (26.0, 30.0)	29.0 (27.0, 30.0)
CSS, median IQR	0.0 (0.0, 6.0)	1.0 (0.0, 3.5)	8.0 (2.0, 11.0)	4.0 (1.5, 9.0)
OSIT-J, median IQR	–	–	2.0 (0.5, 5.5)	2.0 (1.5, 6.0)
LEDD, mg, median IQR	–	–	340.0 (190.0, 555.0)	300 (212.5, 430.0)
Firmicutes/bacteroidetes ratio	0.97	0.72	0.66	0.74

*APP* appendectomy, *PD* Parkinson’s disease, *IQR* interquartile range, *LEDD* levodopa-equivalent daily dose, *OSIT-J* Odor Stick Identification Test for Japanese, *CSS* constipation scoring system, *MMSE* mini-mental state examination, *UPDRS* Unified Parkinson’s Disease Rating Scale, *HY scale* Hoehn & Yahr Scale, *BMI* body mass index.

### Flora genesis results

Cluster classification using the data from all 20 samples revealed that 491 bacterial clusters were classified and 377 clusters (76.8% (overall) of bacterial clusters) were annotated. At each sample level, 62–77% of the clusters of bacterial species were annotated.

### Microbiome profile analysis

**Figure 1:**
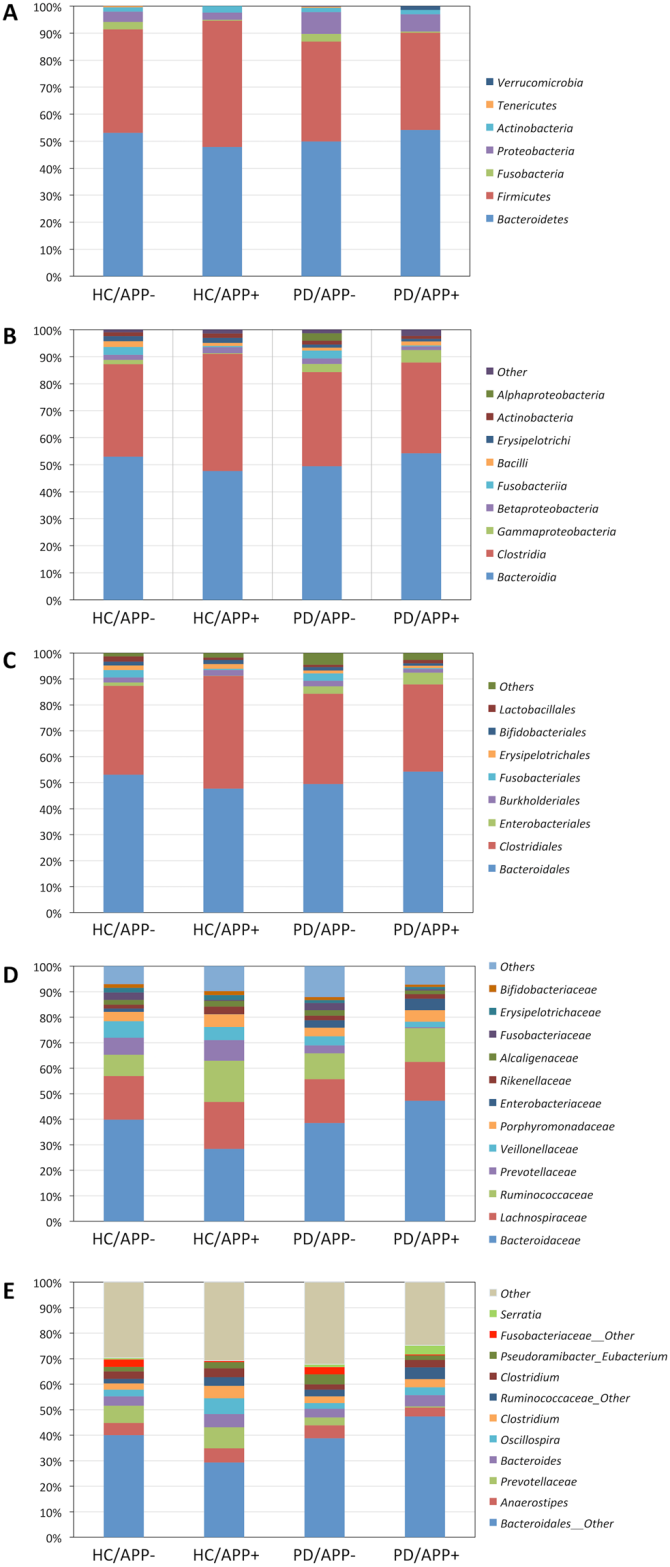
Microbiome profile analyses of average phylogenetic contents. Microbiome profile analyses at the phylum (**A**), class (**B**), order (**C**), family (**D**), and genus levels (**E**). HC/APP−, control without history of appendectomy (APP); HC/APP+, control with history of APP; PD/APP−, patient with PD without a history of APP; PD/APP+, patient with PD with a history of APP.

Microbiome profile analyses of the average phylogenetic subgroups in each group are shown in Fig. 1. Furthermore, complete assigned data of the phylogenic profile of the microbiome are shown in Table e-1.

At the phylum level (Fig. 1A), the two major phyla observed were Bacteroidetes and Firmicutes. The Firmicutes*/*Bacteroidetes ratio did not differ significantly among the four groups (PD/APP+ : 0.66; PD/APP−: 0.74; HC/APP+: 0.97; HC/APP−: 0.72; *p* = 0.337, using the Chi-squared test), barring the HC/APP + groups (Table 1). Proteobacteria were more abundant in patients with PD (PD/APP+ and PD/APP−) than in HCs (HC/APP+ and HC/APP−) (*p* = 0.017, using the Mann–Whitney U test; the same has been used hereinafter). Fusobacteria tended to show smaller ratios in individuals with a history of APP (PD/APP+ and HC/APP+) than in those without (PD/APP− and HC/APP−) (*p* = 0.026).

The two major classes identified were Bacteroidia and Clostridia (Fig. 1B). Gammaproteobacteria were more abundant in patients with PD (PD/APP+ and PD/APP−) than in HCs (HC/APP+ and HC/APP−) (*p* = 0.031). Fusobacteria tended to show smaller ratios in individuals with a history of APP (PD/APP+ and HC/APP+) (*p* = 0.026).

The two major orders detected were Bacteroidales and Clostridiales (Fig. 1C). Enterobacteriales were more abundant in the PD group (PD/APP+ and PD/APP−) than in the control group (HC/APP+ and HC/APP−) (*p* = 0.040). Fusobacteriales tended to show smaller ratios among participants with a history of APP (PD/APP+ and HC/APP+) (*p* = 0.026).

*Bacteroidaceae* (Bacteroidales), *Lachnospiraceae* (Clostridiales), *Ruminococcaceae* (Clostridiales), *Prevotellaceae* (Bacteroidales), *Veillonellaceae* (Clostridiales), and *Porphyromonadaceae* (Bacteroidales) were the major families detected (Fig. 1D). *Enterobacteriaceae* were more abundant in patients with PD (PD/APP+ and PD/APP−) than in HCs (HC/APP+ and HC/APP−) (*p* = 0.040). *Fusobacteriaceae* tended to show smaller ratios in participants with a history of APP (PD/APP+ and HC/APP+) (*p* = 0.047).

Various genera, such as *Anaerostipes* (Bacteroidales) and *Bacteroides* (Clostridiales) were observed (Fig. 1E). The genus *Serratia* was more abundant in patients with PD (PD/APP+ and PD/APP−) than in HCs (HC/APP+ and HC/APP−) (*p* = 0.038). The genera from family *Prevotellaceae* tended to show lower ratios in patients with PD, especially in the PD/APP+ group, and *Fusobacteriaceae* tended to show lower ratios in participants with a history of APP (PD/APP+ and HC/APP+) (*p* = 0.075); however, the difference was not significant.

### Species diversity analysis

**Figure 2:**
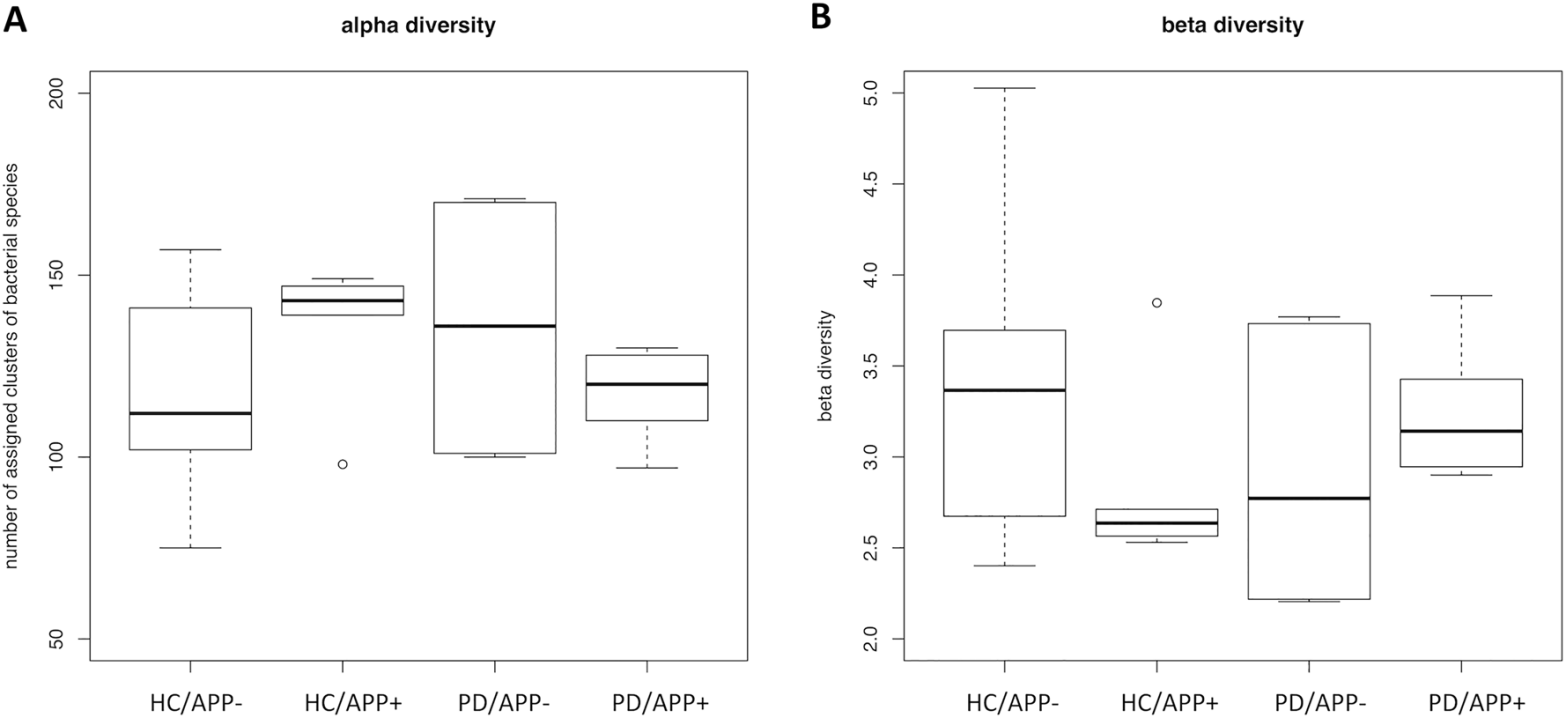
Species diversity analysis. The variances in the alpha and beta diversities of participants without a history of appendectomy (APP) were higher than that in participants with a history of APP. There was no significant difference between the two groups; the *p*-values of the alpha and beta diversities were 0.095 and 0.095, respectively. HC/APP−, control without history of APP; HC/APP+, control with history of APP; PD/APP−, patient with PD without a history of APP; PD/APP+, patient with PD with a history of APP.

After species annotation, the species diversity was investigated based on the number of species assigned clusters. Complete species-assigned results and their statistics are shown in Table e-2. As shown in Fig. 2, the variances in the alpha and beta diversities of the participants with a history of APP (PD/APP+ and HC/APP+) were lower than those of participants without a history of APP (PD/APP− and HC/APP−). Therefore, the difference between HCs and patients with PD was clearer in samples from the PD/APP+ and HC/APP+ groups than in those from the PD/APP− and HC/APP- group; however, the differences were not significant; the *p*-values for the alpha and beta diversities were 0.095 and 0.095, respectively. In the subsequent analyses, further analyses were focused on APP cohort to clarify the difference in composition of HCs and patients with PD in samples from APP+ groups.

**Figure 3:**
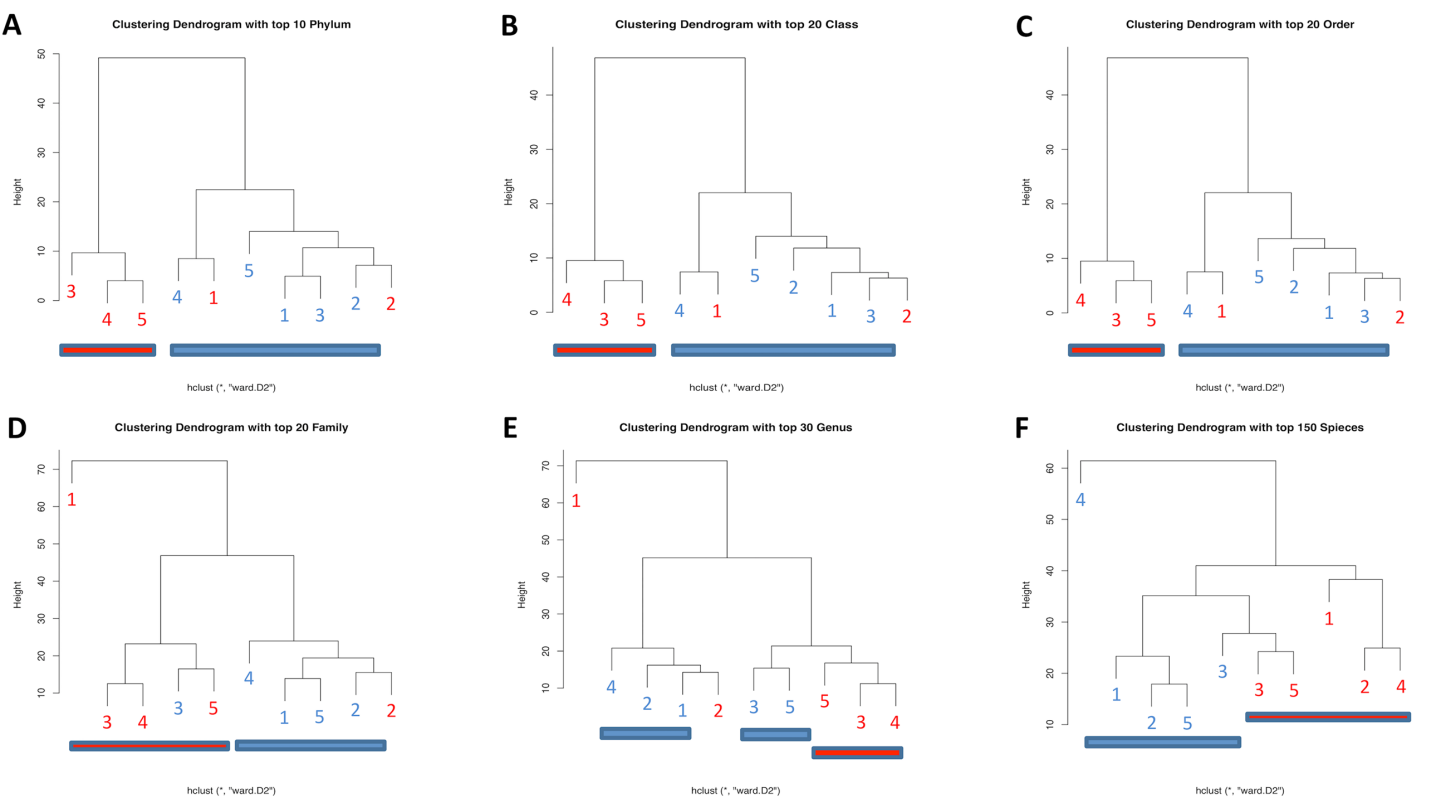
Clustering dendrogram. Hierarchical clustering with top 10 enriched phyla (**A**), top 20 enriched classes (**B**), top 20 enriched orders (**C**), top 20 enriched families (**D**), top 30 enriched genera (**E**), and top 150 enriched species (**F**). Red number, control with history of appendectomy (APP); Blue number, patient with PD with a history of APP.

As we investigated the differences in the diversities between HCs and patients with PD with a history of APP, hierarchical clustering was performed using HC/APP+ and PD/APP+ data. As shown in Fig. 3, in hierarchical clustering with the top 10 enriched phyla, top 20 enriched classes, top 20 enriched orders, top 20 enriched families, top 30 enriched genera, or top 150 enriched species, HC/APP+ and PD/APP+ were separated marginally. Therefore, there were phylogenic differences observed between the two groups. The complete assigned data for the hierarchical clustering of the microbiome are shown in Fig. e.

### UniFrac analysis

**Figure 4:**
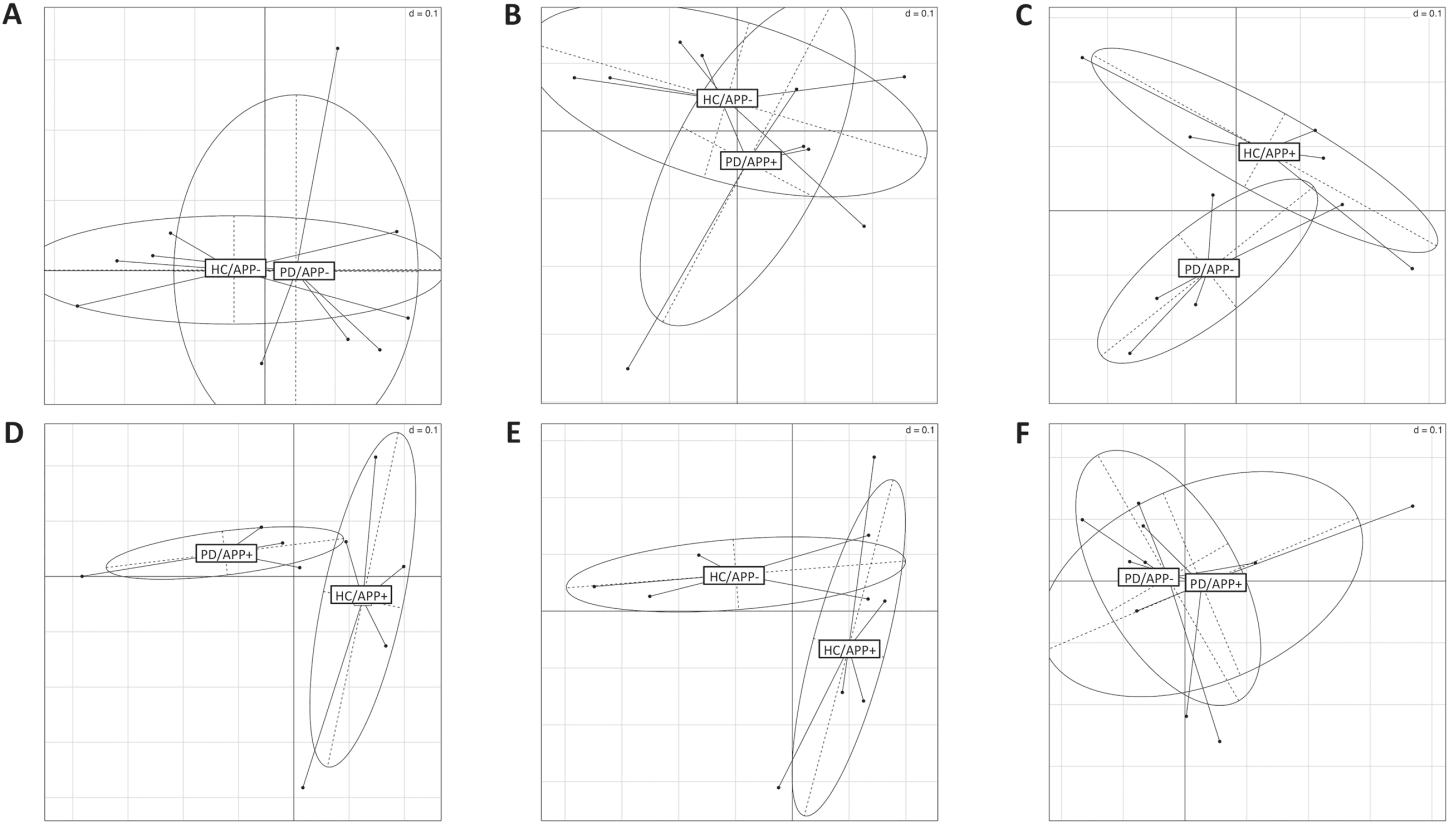
UniFrac analysis. UniFrac analysis between the HC/APP− and PD/APP− (**A**), between the HC/APP− and PD/APP+ (**B**), between the HC/APP+ and PD/APP− (**C**), between the HC/APP+ and PD/APP+ (**D**), between the HC/APP− and HC/APP+ (**E**), and between the PD/APP− and PD/APP+ (**F**). Controls with a history of appendectomy and patients with PD, with clear separation of the two clusters. HC/APP−, control without history of appendectomy (APP); HC/APP+, control with history of APP; PD/APP−, patient with PD without a history of APP; PD/APP+, patient with PD with a history of APP.

UniFrac analysis was performed to clarify the phylogenic differences between the HC/APP + and PD/APP + groups (Fig. 4). In brief, 282 multiple alignment sequences were obtained in the species identification analysis. After a phylogenetic tree was constructed using UPGMA, the tree data were used for the UniFrac analysis. The results of the UniFrac analysis were assessed using PERmutational Multivariate ANalysis Of VAriance (PERMANOVA) and principle coordinates analysis (PCoA) plots. PERMANOVA, also known as non-parametric multivariable ANOVA (MANOVA), was used to evaluate the significant phylogenic differences between each groups. UniFrac analysis revealed that there was significant phylogenic difference between the two groups in the APP cohort (PD/APP+ and HC/APP+; *p* = 0.006 by PERMANOVA after UniFrac analysis) comparing to combinations including the non-APP cohort, such as PD/APP− and HC/APP− (*p* = 0.649 by PERMANOVA after UniFrac analysis). In the PCoA plot, as shown in Fig. 4D, the HC/APP+ and PD/APP+ groups were separated into two distinct clusters, which represented another visualized index of clear differences in the phylogenic profiles between the HC/APP+ and PD/APP+ groups.

## Discussion

In the present study, we found that patients with PD, especially those who had a history of APP, had a more severe form of constipation compared to participants from the other three groups in the analysis of clinical features. We considered that the pattern would be observed in all results, because constipation is one of the most frequent non-motor symptoms in patients with PD^8^. Constipation is a commonly noted feature in prodromal PD and is apparent as early as 20 years before the diagnosis of motor PD^9^. Accordingly, constipation may predict the occurrence of PD^10^.

Certain studies have demonstrated that the gut microbiota of patients with PD shows a low abundance of *Prevotella*^11,12^, whereas other studies have shown contradictory results^13,14^. *Prevotella* has been shown to be present at significantly low levels patients with constipation^15,16^. Our results also indicated that the abundance of *Prevotellaceae* decreased with the severity of constipation. Therefore, the abundance of *Prevotellaceae* in the gut microbiota may be correlated with the severity of constipation, and not with PD.

Several conditions, including obesity^17^, diabetes mellitus type 2^18^, and hypertension^19^, have been linked to an increase in the Firmicutes/Bacteroidetes ratio. Individuals from the HC/APP + group had the highest body mass index. A high Firmicutes/Bacteroidetes ratio in the HC/APP + group may indicate a better nutrient condition than that in the PD group (PD/APP+ and PD/APP−). A pattern of reducing Firmicutes/Bacteroidetes ratio in patients with PD was observed between the PD and control groups in a previous study^14^.

Previous studies have shown that the level of microbes from family *Enterobacteriaceae* was higher in feces samples collected from patients with PD than in those collected from HCs^11,13^. Microbes from the groups Proteobacteria, Gammaproteobacteria, Enterobacteriales, and *Enterobacteriaceae* were more enriched in patients with PD than in HCs. We found that at the genus level, *Serratia* had a higher abundance in feces samples from patients with PD than in those from HCs. *Enterobacteriaceae*, such as *Escherichia coli* and *Salmonella*, produce bacterial amyloids named “curli”^20^. Curli is the major proteinaceous component of enteric biofilms^20^. CsgA is the major structural subunit of curli fibers and a structural constituent of amyloid fibers^21,22^. CsgA can accelerate α-synuclein aggregation and promote motor abnormalities in α-synuclein-overexpressing (ASO) mice^23^. In ASO mice, colonization with *E*. *coli* promoted α-synuclein pathology in the gut and brain^23^. Therefore, *Enterobacteriaceae* may induce an insoluble α-synuclein pathology. Furthermore, a state of increased intestinal permeability (i.e., leaky gut) has been observed in patients with PD^24^. Increased intestinal permeability and staining specific for *E*. *coli* significantly correlated with α-synuclein staining in patients with PD but not in controls^25^.

Reportedly, intestinal permeability and neutrophil activity are closely linked to the pathophysiology of inflammatory bowel disease (IBD)^26^. In a bacterial culture of biopsies from ileum and ascending and sigmoid colon collected from patients with IBD, *Enterobacteriaceae* (primarily *E. coli*) and *Bacteroides* were the predominant aerobes and anaerobes, respectively^27^. *E. coli*, particularly the adherent-invasive *E. coli* pathotype, has been implicated in the pathogenesis of IBD^28^. Patients with IBD have a higher incidence of PD than individuals without IBD^29^. Therefore, the intestinal permeability and *Enterobacteriaceae* abundance might influence the progression of PD and IBD. Fecal microbial transplantation has shown efficacy in IBD treatment^30,31^. The gut microbiota from patients with PD was shown to enhance motor dysfunction in mice^32^. A preliminary study indicated that fecal microbiota transplantation can relieve the motor and non-motor symptoms in patients with PD^33^. Therefore, fecal microbiota transplantation may also be effective for treating PD.

In this study, the alpha and beta diversities did not differ significantly between the groups, confirming the findings from other studies^34,35^. However, the variances in the alpha and beta diversities of participants with a history of APP were lower than that in participants without a history of APP. At an early time point after APP, feces samples collected from mice showed diverse patterns of microbial communities, whereas the samples from sham-operated mice showed a microbial pattern that was highly homogeneous^7,36^. These differences in the microbial composition are thought to correlate with the abundance of IgA^+^ cells. The number of IgA^+^ cells decreased markedly in the mice at an early time point (2–4 weeks) after APP, but not at a late time point (8 weeks)^7^. The bacterial groups present in mice at 8 weeks after APP were similar to that present in sham-operated mice^7^. The appendix has a more diverse microbial composition than the intestine and also considerable interindividual variation^49^. Bacteria from the phylum Fusobacteria might be part of the normal appendix flora, however, their abundance increases in certain cases of appendicitis^37,38^. Our findings of increased *Fusobacteria* in the fecal microbiota of individuals who had not undergone APP are suggested to be due to microbial leakage in the appendix. Therefore, the fecal microbiota in patients without a history of APP may exhibit considerable interindividual variation with greater variances in alpha and beta diversities. Hierarchical clustering revealed that there were some differences between the HCs and patients with PD with a history of APP. For example, in patients with PD, the abundances of *Proteobacteria*, *Gammaproteobacteria*, *Serratia*, and *Enterobacteriales* increased at the phylum, class, order, and genus levels, respectively. In the APP cohort, the abundances of *Fusobacteria*, *Fusobacteriia*, *Fusobacteriales*, and *Fusobacteriaceae* decreased at the phylum, class, order, and family levels, respectively. Furthermore, the UniFrac analysis revealed that there was a significant phylogenic difference between the microbes present in HCs and those present in patients with PD with a history of APP, and these two groups were separated into two distinct clusters. These results suggest the correlation between gut microbiota and PD in patients who have undergone APP.

The important limitations of the present study include the small samples size and the lack of pathological confirmation. Although APP leads to certain variations in the gut microbial composition, it differs considerably from that observed in patients with PD. Therefore, it remains unclear if the changes induced in the gut microbial composition upon APP correlate with those observed in patients with PD. This is a preliminary study to elucidate the role of the appendix in the development of PD based on the gut microbiota. We will continue our investigation with a much larger sample size. We will use the framework established in the present study as a basis for future research on the association between gut microbiota and the appendix in PD. It will be necessary to increase the sample size in order to proceed.

In conclusion, a significant phylogenic difference was observed in the fecal microbial composition between HCs and patients with PD who had undergone APP. However, the abundance of microbes from the family *Enterobacteriaceae* was higher in feces samples from patients with PD compared to that in samples collected from HCs, irrespective of whether the individual had undergone APP. We suggest the following hypothesis for the induction of PD: First, *Enterobacteriaceae* induce an insoluble α-synuclein pathology in the intestine. Second, the vermiform appendix harbors a part of insoluble α-synuclein pathology. The insoluble α-synuclein is then transported from the gut to the brain via the vagal nerve. Eventually, the insoluble α-synuclein spreads to the brain. However, further studies are required to confirm this hypothesis.

## Materials and methods

### Participants and ethical standards

In this prospective cohort study, we compared the gut microbiome composition in patients with PD and HCs with or without a history of APP. PD was diagnosed based on the guidelines of the United Kingdom Parkinson’s Disease Society Brain Bank clinical diagnostic criteria^39^. We excluded patients with PD receiving device-aided therapy. We selected HCs from among individuals without any signs of parkinsonism with age- and sex-matched controls. The participants were then divided into the following four categories based on the presence or absence of APP history: PD/APP+, PD/APP−, HC/APP+, and HC/APP−. Five participants were included in each group (twenty participants in total).

All procedures performed in the studies involving human participants were in accordance with the ethical standards of the institutional ethics committee as well as the guidelines of the 1964 Helsinki declaration and its later amendments or comparable ethical standards (permit number: 1146). We received written informed consent of all participants.

### Clinical data

We evaluated disease severity using the HY scale^40^ and the UPDRS^41^. We also calculated a LEDD to account for the dopaminergic agents administered to each patient with PD in the PD/APP+ and PD/APP− groups using previously published or available dosage data as well as the formula proposed by Tomlinson et al.^42^. Cognitive function was assessed using the MMSE^43^. The degree of constipation was quantified using the CSS^44^. The OSIT-J was used to evaluate olfaction^45^. Assessments using the MMSE, CSS, and OSIT-J were based on medical interviews and examinations performed by the authors. Details of the antiparkinsonian treatment were recorded, and the total daily dose of levodopa was calculated for each patient with PD.

### DNA amplicon sequencing and operational taxonomic unit (OTU) assignment

Twenty microbial DNA samples (0.83 ± 0.72 (mean ± SD) µg in each sample) were extracted from the fecal samples of the participants using the Extrap Soil DNA Kit Plus ver.2 (NIPPON STEEL & SUMIKIN Eco-Tech Corporation, Chiba, Japan). A sufficient quantity of DNA sample was obtained from all 20 samples used for analysis. Amplification of the 16S rRNA gene amplicon was confirmed using standard 25-cycle PCR. Sequencing analysis was performed using the amplicons. The V1–V2 region of the 16S rRNA genes was amplified and sequenced using the MiSeq system with the MiSeq Reagent Kit v3 (Illumina, San Diego, CA, USA) according to the manufacturer's instructions.

The sequence data were preprocessed and analyzed using the "Flora Genesis software" (Repertoire Genesis Inc., Ibaraki, Osaka, Japan). In brief, the R1 and R2 read pairs were joined and the chimera sequences were removed. The OTUs were assigned using the open-reference method with the 97% ID prefiltered Greengenes database (http://greengenes.secondgenome.com)^46^ and the UCLUST algorithm^47^. The representative sequences of each OTU were selected, and the taxonomy was assigned using the Ribosomal Database Project (RDP) classifier^48^ with a threshold score of ≥ 0.5. OTUs with the same annotation were grouped regardless of the RDP score.

### Statistics

Statistical analyses were performed using R version 3.6.1. Statistical tests were performed using the Mann–Whitney U test. Hierarchical clustering was performed by analyzing the euclidean distance (dist (x, method = "euclidean")) and using the Ward's method (hclust (x, method = "ward.D2")). A *p* value of less than 0.05 was considered to be statistically significant.

### Species diversity

Species diversity was assessed based on the alpha and beta diversities^49^. Alpha diversity, which indicates the diversity within each group, was represented by the number of species in each sample. Beta diversity, indicating a comparison of the diversity between groups, was calculated as follows: beta diversity = gamma diversity/alpha diversity, where gamma diversity was represented by the overall diversity in all samples.

### UniFrac analysis

UniFrac analysis^50^ was performed using GUniFrac (otu.tab, tree, alpha = c (0, 0.5, 1)) provided with the R package GUniFrac_1.1. In brief, tree data were obtained from the bacterial species analysis data. Singleton data were omitted from the bacterial species analysis data, and sequence data were obtained using BioPerl ver1.006924 and Perl ver5.16.3. Multiple alignments were performed using the sequence data with ClustalW 2.1, and the data with outlier scores were omitted. Multiple alignment data were used to construct a tree using the R package ape_5.3. Otu.tab data were obtained using the Rarefy function in the GUniFrac package. The PERMANOVA and PCoA plots were also constructed using the UniFrac data.

### Ethics approval and consent to participate

All procedures performed in the studies involving human participants were in accordance with the ethical standards of the institutional ethics committee as well as the guidelines of the 1964 Helsinki declaration and its later amendments. In addition, this study was approved by the ethical committee of Kumamoto University (permit number: 1146).

## Supplementary Information

Supplementary Information 1.

Supplementary Information 2.

Supplementary Information 3.

Supplementary Information 4.

## Data Availability

Anonymized data will be shared by request from any qualified investigator. All data generated or analysed during this study are included in this published article. The nucleotide sequence data reported are available in the DDBJ Sequenced Read Archive under the accession numbers DRX197966 and DRX197985.

## Abbreviations

PD: Parkinson’s disease
APP: Appendectomy
HC: Healthy control
CNS: Central nerves system
APP: Appendectomy
CSS: Constipation scoring system
HY: Hoehn & Yahr
UPDRS: Unified Parkinson’s Disease Rating Scale
MMSE: Mini-mental state examination
OSIT-J: Odor Stick Identification Test for Japanese
LEDD: Levodopa-equivalent daily dose
PERMANOVA: PERmutational Multivariate ANalysis Of Variance
PCoA: Principle coordinates analysis
ASO: α-Synuclein overexpression
IBD: Inflammatory bowel disease
OTU: Operational taxonomic unit
RDP: Ribosomal Database Project
