# Supplementary figures and images for: Gut microbiota of Parkinson’s disease in an appendectomy cohort: a preliminary study
Source: Sci Rep. 2023 Feb 7;13:2210. doi: 10.1038/s41598-023-29219-2 (PMC9905566; doi:10.1038/s41598-023-29219-2)

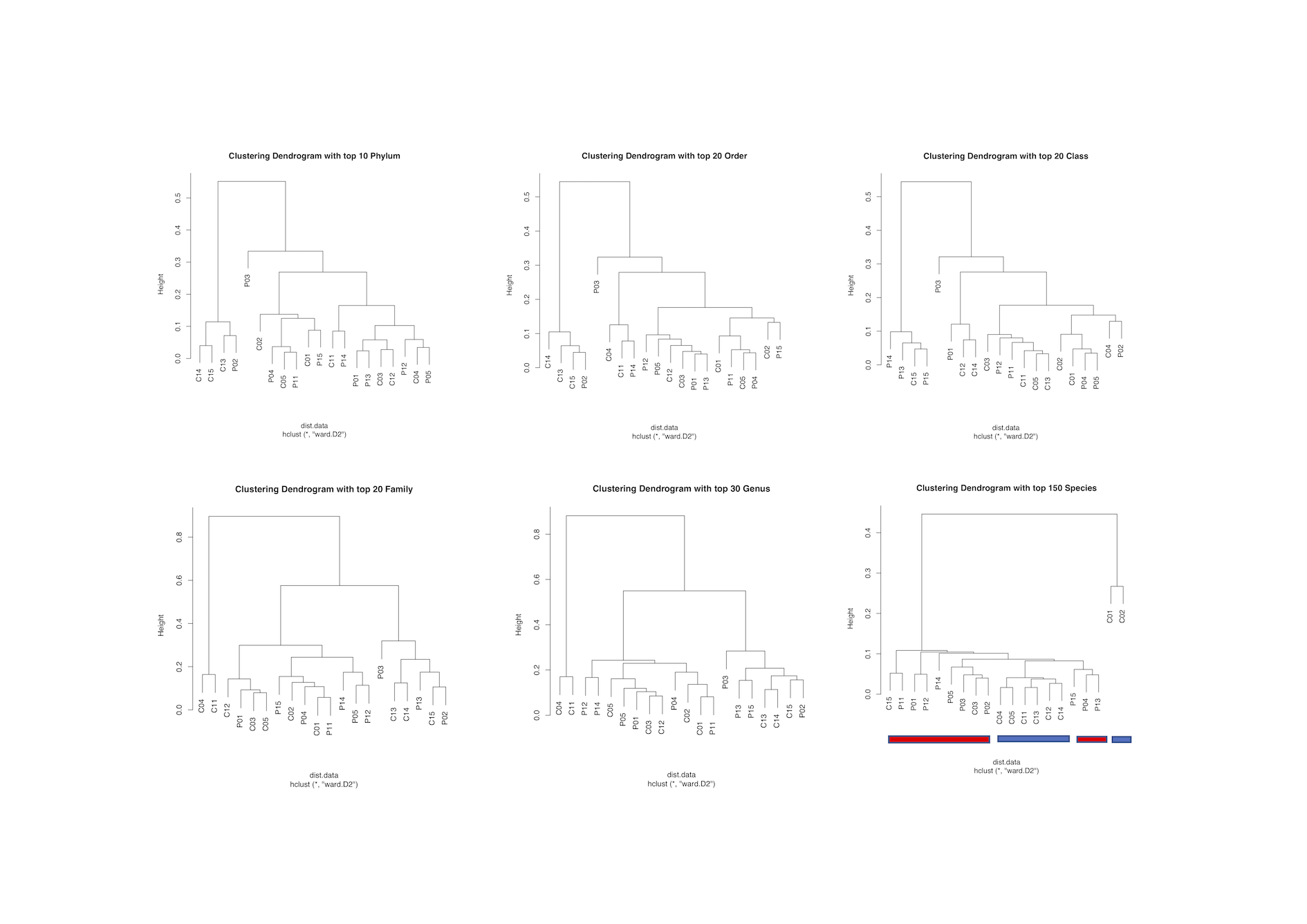

Supplement: Supplementary file 1 — Supplementary Information 1. [file 41598_2023_29219_MOESM1_ESM.tiff]
