# Supplementary material for: Gut microbiota of Parkinson’s disease in an appendectomy cohort: a preliminary study
Source: Sci Rep. 2023 Feb 7;13:2210. doi: 10.1038/s41598-023-29219-2 (PMC9905566; doi:10.1038/s41598-023-29219-2)
Supplement: Supplementary file 2 — Supplementary Information 2. [file 41598_2023_29219_MOESM2_ESM.docx]

**Figure legends**

**Figure e: Clustering dendrogram for fully assigned data**

Hierarchical clustering analysis of all subjects showed that it was difficult to separate clusters at the level of phylum, order, class, family, and genus for the presence or absence of Parkinson's disease. However, it was possible to separate them at the species level.

HC/APP-: C01-C05, HC/APP+: C11~C15, PD/APP-: P01-P05, PD/APP+: P11-P15

.
